# Commentary: Making Patient Data Count—Opportunities and Challenges for Open Science in Clinical Psychology

**DOI:** 10.1002/jclp.70162

**Published:** 2026-06-05

**Authors:** Jan C. Cwik, Barbara Cludius, Dorothée Bentz, Marcel Riehle, Johannes C. Ehrenthal, Anke Haberkamp, Annika Clamor, Simon E. Blackwell, Maike Salazar Kämpf, Edgar Nazarenus, Rima‐Maria Rahal, Anne Möllmann, Jakob Fink‐Lamotte, Juliane Burghardt, Jan R. Boehnke, Kevin Hilbert

**Affiliations:** ^1^ Professorship for Applied Psychology, Faculty of Health Care Hochschule Niederrhein Krefeld Germany; ^2^ Department of Psychology University of Cologne Cologne Germany; ^3^ Department of Psychology University of Bremen Bremen Germany; ^4^ Department of Biomedicine and Faculty of Psychology University of Basel Basel Switzerland; ^5^ Department of Psychology University of Witten/Herdecke Witten Germany; ^6^ Faculty of Human Sciences MSH Medical School Hamburg Hamburg Germany; ^7^ Institute of Psychology University of Göttingen Göttingen Germany; ^8^ Faculty of Psychology Technische Universität Dresden Dresden Germany; ^9^ Department of Experimental Psychopathology University of Hildesheim Hildesheim Germany; ^10^ Behavioral Law & Economics Max Planck Institute for Research on Collective Goods Bonn Germany; ^11^ Clinical Psychology University of Potsdam Potsdam Germany; ^12^ Division of Clinical Psychology Karl Landsteiner University of Health Sciences Krems Austria; ^13^ School of Health Sciences University of Dundee Dundee UK; ^14^ Faculty of Health Sciences Health and Medical University Erfurt Erfurt Germany

## Abstract

Open science practices—such as preregistration, data and material sharing, and open‐access dissemination—are increasingly promoted across psychology, yet their specific value for clinical psychology has often been overlooked. This commentary argues that open science is particularly crucial for clinical psychology, where studies rely on small, hard‐to‐recruit patient samples, ethically sensitive data, and complex psychotherapeutic interventions. However, applying open science practices in one's research can be challenging. And yet, it is becoming increasingly necessary to take a stance on this. To support our colleagues in clinical psychology to take a stance on open science practices and to motivate them to apply these practices in their research, we describe here how the use of open science practices benefits or can benefit clinical psychology science and practice and provide some perspective on why we believe that the increasing use of open science practices is consistent with good scientific practice and the ethical standards of our profession. In addition, we discuss how calls for increased implementation of open science practices in psychological research can be reconciled with some of the challenges, concerns, and conflicts that can arise around open science practices, especially in clinical psychology. Finally, we extend open science's inclusive and collaborative stance to include “experts by experience” in the research process. Including our research topics in the research process is a specific facet of the open science approach in clinical psychology that has been overlooked.

1

Open science practices are increasingly gaining traction. While there is no single definition of open science, it is mainly understood as an umbrella term for practices that make research more accessible and more transparent. Common practices include sharing data, materials, or code, the pre‐registration of planned analyses, and making research results available as preprints and open‐access publications (Center for Open Science, https://www.cos.io/open-science). There are proposals to use open science practices as professional recruitment and promotion criteria in psychology (Schönbrodt et al. 2022; Gärtner et al. 2022; Fink‐Lamotte 2024). However, various aspects of open science are particularly important and should be implemented more specifically in clinical psychology. Despite a relatively long history of pre‐registration of clinical trials, other aspects of open science are less commonly used. For example, data provision on open science platforms is rather the exception than the norm (Tackett et al. 2019). Both methodological aspects (e.g., small or unstructured datasets or qualitative data) as well as data sensitivity are obstacles to using open science practices in clinical psychology (Walsh et al. 2018). However, these obstacles make it even more important to implement open science practices so that these sensitive data, which are often only available in small quantities and require high levels of effort to gather, can be optimally used to contribute to a better understanding of mental health (Walsh et al. 2018).

This commentary argues that open science practices are particularly crucial for clinical psychology, where data are often scarce, heterogeneous, and ethically sensitive. We outline the specific opportunities and barriers for adopting open practices in this field. Furthermore, this commentary illustrates to scientists in clinical psychology, at all career stages, how the use of open science practices benefits or may benefit clinical psychological science and practice. This can be, for example, by increasing the robustness and quality of research findings and the cost‐effectiveness of using resources, by stimulating innovation, and eventually by progress in improving evidence‐based treatments across age groups and mental health conditions. Furthermore, we discuss how calls for increased implementation of open science practices in psychology research can be reconciled with some of the challenges, concerns, and conflicts that can arise concerning open science practices in clinical psychology. We hope to inspire other researchers to make greater use of open science practices.

While this article aims to convey and discuss a stance on open science practices in clinical psychology, it is not intended to serve as a concrete guide to implementing them. A separate manuscript will be created for this purpose, outlining the step‐by‐step implementation of open science practices in more detail elsewhere (Salazar Kämpf et al. 2025). Several of the arguments outlined in this article will be familiar to psychology researchers who are well‐versed in open science practices and may not seem novel or specific to clinical psychology. However, the relatively slow uptake of open science practices by many clinical researchers suggests that they believe these general points are somehow not applicable to the clinical field. Hence, while this article does consider some “special cases” more specific to clinical psychology, it also covers some of the broader principles; clinical researchers need to hear that these principles are equally applicable to their work, too, even if their implementation sometimes requires special considerations.

In this commentary, we focus particularly on three core pillars that are especially salient for clinical psychology: (1) preregistration and Registered Reports as tools to strengthen confirmatory rigor in complex clinical designs; (2) responsible data and material sharing under appropriate governance structures that account for the sensitivity of clinical data; and (3) analytic transparency, including documentation of analytic decisions and qualitative research practices. By structuring our discussion around these pillars, we aim to illustrate how general open science principles can be meaningfully adapted to the practical and ethical realities of clinical research.

## 2 The Case for Open Science Practices in Clinical Psychology

(Clinical) Psychological research faces various major challenges. These include underspecified theories (e.g., learned helplessness theory; Burghardt and Bodansky 2021), low measurement accuracy, the lack of robustness of research results, and duplicate research projects (Niemeyer et al. 2023; Tackett et al. 2019; Tackett et al. 2019). These challenges are especially relevant because of the resource‐intensive nature of data collection in clinical research and the high social relevance of rapid improvements. Open science practices can contribute to reducing some of these challenges.

### 2.1 Promoting the Quality and Robustness of Research

One advantage of open science is that it can help to increase the quality and robustness of clinical psychological research. Many studies have shown that the results in psychology are not robust and cannot be (fully) replicated. This also appears to apply to clinical psychology, where a replication score of 44% was estimated (Youyou et al. 2023). This means research efforts are invested in research questions that may not be worth pursuing. As a result, fewer resources are available to generate evidence for effective treatments.

The replication crisis is fueled by various factors, some particularly relevant in clinical psychology. Examples include: (1) high flexibility in data analyses (Simmons et al. 2011), which is amplified in large‐scale studies with various covariates, such as clinical trials; (2) a lack of standardization in methods and materials, particularly due to not reporting certain characteristics (Brown et al. 2014); (3) statistical reporting errors (Nuijten et al. 2016); (4) questionable research practices, including selective reporting and hypothesizing after results are known (HARKing), which can inflate confirmatory analyses with exploratory analyses (Murphy and Aguinis 2019); (5) allegiance effects by researchers and by therapists, who conduct the treatment (i.e., researchers or therapists believe that the psychotherapeutic treatment they developed or use is superior to other treatments) (Leichsenring et al. 2017); and (6) underspecified theories and constructs (Burghardt and Bodansky 2021).

Open science practices are one way to address the above‐mentioned problems and thus enhance the probability of replicating research results (Shrout and Rodgers 2018; Tackett et al. 2019). Open science practices include pre‐registration, open materials, and open data (Tackett et al. 2019). First, pre‐registration implies determining hypotheses, methods, and analysis plans a priori and sometimes documenting the rationale behind these decisions. Pre‐registration creates transparency about what analyses were planned a priori, which limits HARKing and promotes the likelihood of agreeing on one analysis plan, which reduces p‐hacking (the practice of manipulating data analysis or experimental design to achieve statistically significant results, often by selectively reporting outcomes or adjusting parameters). At the same time, pre‐registration proves that p‐hacking was not used while still allowing for explorative analyses, provided that these are properly flagged. It can also reduce statistical reporting errors and questionable research practices (Petersen et al. 2022; Tackett et al. 2019). Further, it can enhance the quality of conducted studies by fostering more in‐depth engagement with the project before running the study. However, it is worth noting that pre‐registration reduces flexibility but does not guarantee that the researchers will publish their results. Lakens et al. (2024) showed that many preregistered studies remain unpublished. Addressing the ‘file drawer problem’ requires changes in editorial policies and incentive structures, not only the behavior of individual researchers. Concretely, journals in clinical psychology could encourage or require preregistration for confirmatory trials, offer Registered Report format that provides in‐principle acceptance independent of study outcomes, and implement mandatory data‐availability statements that transparently document whether and how data can be accessed. Explicit editorial policies affirming the scientific value of well‐conducted studies with null findings may further reduce publication bias. Such structural measures can help align individual researcher incentives with collective scientific goals. Second, open materials allow fellow researchers to thoroughly evaluate an original study, facilitate the standardization of research, and therefore increase replicability (Shrout and Rodgers 2018). As a result, studies could be created more effectively. In clinical psychology, this could reduce allegiance effects. When researchers and therapists who did not conduct a study themselves have access to detailed information about the original research, including, for example, clinical manuals used in intervention studies or instructions used in experimental studies, they can more faithfully implement the examined interventions, methods, and attitudes (Leichsenring et al. 2017; Tackett et al. 2019). Third, open data allows us to verify the reported results. And last, but not least, open research practices have the potential to positively influence research culture in the long term.

Overall, while open science practices may slow down research efforts in the beginning and need additional resources for planning and pre‐registering the study, as well as for data and material sharing, they bear the promise of leading to more robust and higher‐quality research findings by at least reducing the use of questionable research practices. Thus, it may lead to more efficient research efforts since less time is spent on spurious findings. In addition, the formulation of central theoretical and methodological aspects already developed for the pre‐registration can speed up the writing‐up of completed studies.

### 2.2 Reducing Duplication of Simultaneous Research Efforts and Increasing Resources for High‐Quality Studies

Another advantage of open science is that it can reduce simultaneous research efforts and free up resources for high‐quality studies. Clinical trials are very costly due to the challenges of recruiting the sample. Therefore, it is unsurprising that most clinical trials do not include sample sizes to detect small effect sizes (Reardon et al. 2019; Bakker et al. 2012) or a clinically relevant difference (Cuijpers 2016). When several studies on similar phenomena are conducted simultaneously, because researchers are unaware of each other's projects, this ties up capacities and resources that could be used more effectively when combined. The systematic costs for the scientific community of not sharing resources are immense (see United Nations Educational, Scientific and Cultural Organization 2021). Duplication costs for recreating studies stashed away in private filing systems or rerunning simultaneous experiments without knowing about other attempts run a staggering tally. In effect, scientific insight itself is stalled because resources that could be devoted to driving insight and innovation are swallowed by attempts to patch the shaky foundation on which research is built. This effect may be particularly pronounced in clinical psychology, where data often have to be collected at great expense and over a long period of time, for example, because events and populations are rare (e.g., suicidality, autism) or because the treatments delivered in the study are complex.

Open science practices should enable transparency around previous findings. This aims to avoid repeating research on ideas with unpublished null or published false positive results, conserving resources by acknowledging these outcomes upfront. Open science practices, especially pre‐registrations and open peer reviews, should help build on previous findings based on reliable data. Furthermore, rather than conducting separate studies simultaneously, engaging in multi‐site collaborations is promising for the future of clinical psychology science. These multi‐site collaborations can be made possible by open science and can contribute to research efforts that could be combined to conduct larger‐scale clinical trials. Large‐scale collaborations such as the Psychological Science Accelerator (Moshontz et al. 2018) demonstrate how coordinated efforts across sites can increase power and generalizability—an approach particularly valuable for clinical trials with rare or heterogeneous patient populations. Additionally, resources could be invested in conducting more prospective studies, which have been called for in various domains, to assess risk and maintain psychopathology factors (e.g., Lincoln et al. 2022). This is especially important since prospective studies may lead to vastly different findings than retrospective studies. For example, the lifetime prevalence of mental disorders was twice as high in prospective studies compared to retrospective studies (Moffitt et al. 2010).

### 2.3 Fueling Innovation and Improving Dissemination by Promoting an Open and Direct Scientific Discourse

A further advantage of open science is that it encourages an open and direct scientific discourse, which fosters innovation and helps to improve the dissemination of research results. Gold (2021) has argued that research has become less productive over the last century and that innovation comes at higher costs. In part, this decreased efficiency is proposed to stem from a strong emphasis on intellectual property, because knowledge is kept within the team and sharing information comes at high costs (Gold 2021). This may be the case particularly for clinical psychology, given the many events and populations with low prevalence (e.g., suicidality, autism): here, free access to data can help enable more robust results from larger samples by combining data from several research centers. Especially in fields with small populations, such as research on individuals with suicide attempts, sharing data can help identify risk factors (Carpenter and Law 2021; Kirtley et al. 2022).

One example of open science practices providing real value for translational research is the field of oncology (Doroshow and Kummar 2014). Here, significant development was fueled by a national strategic plan implemented by the United States of America to encourage scientists to publish open data and work on open science structures, such as establishing shared data priorities and promoting innovative open data solutions. This open science initiative hoped to decipher the code of cancer by sharing data as extensively as possible since a collective analytic view could reveal patterns inaccessible to isolated research laboratories (Hesse 2018). Another example is the sharing of relevant data at the beginning of the SARS‐CoV‐2 pandemic (Corman et al. 2020). Through open science practices, the development and validation of a real‐time RT‐PCR test for detecting the novel coronavirus (2019‐nCoV) was ensured. This allowed for the creation of a testing method with high sensitivity and specificity, enabling a rapid response to the health crisis.

Open science could be important in disseminating science into clinical practice. For example, one area specific to clinical psychological research is diagnostics. Misdiagnoses due to overestimation or underestimation of symptoms or incorrect judgments regarding the diagnostic classification of symptoms can lead to distortions in a scientific context, e.g., concerning epidemiological data, while in a practical context, they can lead to incorrect treatment (Tackett et al. 2019). To reduce the likelihood of misdiagnoses and thus support clinicians in selecting the appropriate interventions based on the correct diagnosis, appropriate diagnostic instruments should be used (Cwik and Teismann 2017; Wolkenstein et al. 2011). This applies to both clinical psychological practice and clinical psychological research. However, it is still not common for diagnostic instruments to be used standardly in practice (Bruchmüller et al. 2011). The open science movement in clinical psychology and psychotherapy should not only aim to provide data but should also focus on making diagnostic tools, such as questionnaires and diagnostic interviews, available as open material for research and practice (see e.g., Margraf et al. 2017).

## 3 Dealing With Conflicting Interests Arising From Open Science Practices

The research process creates situations in which individual researchers' interests conflict with what would benefit the scientific community. This potential trade‐off between individual and collective interests defines social dilemmas (Corten 2019; Linek et al. 2017). Researchers might fear that open science will increase their costs in favor of benefits for the scientific community, at best. In the traditional research landscape, individual researchers' short‐term self‐interest is often to keep resources (e.g., materials, data, or code) private. There are many reasons why it may appear rational for individuals to keep resources private, including the time and energy it takes to make resources shareable (e.g., formatting, creating meta‐data, writing a codebook), fear of backlash should inconsistencies or errors be uncovered (e.g., coding errors, rounding errors), and preserving one's unique research niche (e.g., slowing competitors' progress). Additionally, some may think that sharing resources gives other researchers a carte blanche for freeriding (e.g., by using only freely available datasets without contributing datasets oneself, see Alós‐Ferrer and Yechiam 2020). This reasoning is exasperated by the competitive and heavily reputation‐based nature of academia. However, because researchers' contributions to the system tend to be repeated (not one‐shot), we may see that others retaliate against non‐cooperation by choosing not to share resources, such that a norm of non‐cooperation (i.e., not sharing resources) is established. Furthermore, some researchers perceive that researchers who forgo open practices may appear more productive, while those who embrace them put in extra effort. Overcoming this dilemma can be resolved by systematic rewards such as data citations, recognition of open practices in hiring and funding decisions, and collective norms (Curry et al. 2020).

Open science can be a tool to help actors in this system establish a norm of cooperation and refocus on common goals like research progress and knowledge generation. On the one hand, construing individuals' research contributions as cooperative contributions to a public good in itself may help researchers assess which norms they find important in this context. In the example of sharing resources, such reflections may yield the insight that sharing data, code, and materials is a socially desirable behavior, especially when research is publicly funded (as is the case for most scientific research at European universities). When potential or perceived costs of cooperation are lowered (Linek et al. 2017), e.g., through providing training or easy‐to‐use guidelines for data‐sharing practices, cooperation rates may rise, and open science practices may be implemented more. Crucially, open science is not all‐or‐nothing—each small step taken towards increasing openness is valuable. It is, therefore, already valuable to take steps that are not necessarily associated with major costs in a given project.

On the other hand, reassessing perceptions of or changing systematic incentives that have traditionally fostered non‐cooperation may provide a way to align an individual's behavior with the collective interest (e.g., Wagenmakers and Dutilh 2016, regarding selfish reasons to use pre‐registration). In the example of sharing resources such as data, code, or materials, incentives like earning citations for data publications, awards for outstanding resource contributions, or prerequisites for receiving funding (Acord and Harley 2013; Tenopir et al. 2011), as well as requirements for hiring (Gärtner et al. 2022) may prove useful for boosting cooperative behavior. In addition, individuals' reputational benefits (e.g., Hardy and Van Vught 2006) may ensure long‐term cooperative contributions. Engaging in open science practices further offers several benefits for individual researchers, including a potential boost in citation counts (see discussion in Langham‐Putrow et al. (2021) regarding open access) or the ability to obtain in‐principle acceptances at academic journals independently of experimental results with Registered Report publications (see Chambers and Tzavella 2022). Recent developments that demonstrate that open science practices are increasingly rewarded and or even demanded, e.g., in appointment procedures or during funding acquisition, reduce the social dilemma. Considering this, open science practices benefit not only competing researchers and the overall research community but also the individual practicing open science.

Taken together, open science can be a tool to increase the robustness of research findings, increase the quality of research by reducing duplication and increasing cooperation to make large‐scale projects possible, and make research more efficient due to quicker and more in‐depth sharing of information and data. However, many researchers in clinical psychology are still reluctant to openly share data or material due to conflicting interests. In the following section, we will outline some of those reservations and present ideas for addressing them.

## 4 Perspectives That Could Be Helpful to Move Individual Behavior and the Field of Clinical Psychology Toward Open Science

### 4.1 The Ethical Obligation to Make Patient Data Count

Clinical data in psychology are often highly sensitive, particularly when they include detailed clinical interviews, longitudinal assessments, or psychotherapy process data. In psychotherapy research, for example, raw session transcripts or audio files contain rich biographical information, references to third parties, and context‐specific details that can create an unusually high risk of re‐identification, particularly in small or specialized clinical populations—even after direct identifiers are removed. These features make unrestricted public sharing of such materials ethically inappropriate in most cases. To our knowledge, large‐scale open sharing of full psychotherapy transcripts is currently rare due to the sensitivity of these materials, reflecting legitimate ethical and legal constraints. Our example is therefore intended to illustrate a conceptual governance model rather than established routine practice.

Responsible open science in clinical psychology must therefore begin with the protection of participants. Several mechanisms can mitigate risks while preserving scientific value. These include sharing synthetic datasets, aggregated statistics (e.g., means, standard deviations, covariance matrices), or anonymized coding schemes rather than raw transcripts. Controlled‐access repositories, data use agreements, and governance procedures for evaluating secondary data requests provide further safeguards. Additionally, consent procedures can transparently inform participants about different levels of data sharing, including the option to restrict commercial use.

At the same time, many patients participate in clinical research with the explicit intention of contributing to scientific progress and improving care for others. Participation in clinical trials often involves considerable time, emotional effort, and the disclosure of highly personal information. Empirical findings suggest that patients are frequently supportive of data reuse for research purposes when adequate safeguards are in place (Richter et al. 2024). Failing to make meaningful scientific use of carefully collected clinical data—particularly when participants have consented to such use—may therefore also raise ethical concerns, as it limits the societal value of their contribution (Rockhold et al. 2019).

From this perspective, open science in clinical psychology is not about unconditional openness, but about balancing two ethical commitments: protecting participants from harm and honoring their willingness to contribute to cumulative knowledge. When implemented within appropriate governance structures, responsible data sharing can serve both goals simultaneously and help ensure that patient data truly “count” for advancing clinical psychological science and improving evidence‐based care.

### 4.2 Viewing Negative Findings as Valuable Research Results on Their Own

As in other research areas, there is a bias in clinical psychology and psychotherapy due to the predominant publication of positive results (Tackett et al. 2017). Null or negative results are published much less frequently (Trespidi et al. 2011). However, the publication of such results is also important to prevent ineffective or even harmful interventions (Trespidi et al. 2011), as shown, for example, by the results of a study on debriefing as a group intervention after major incidents (Rose et al. 2003). Including unpublished data in the overall evaluation of the effectiveness of interventions can lead to a different result than if one only looks at the published data (Whittington et al. 2004). In addition, the various parties involved in the publication process may have negative attitudes towards null results: while researchers may interpret null results or observations of negative effects as a kind of failure (see Iwachiw et al. 2019), there is also an impression among researchers that reviewers and editors are more critical of null results (Casey 2021)—although there is little empirical data on the latter assumption. This publication practice can encourage a publication bias, which can lead to an overestimation of the effectiveness of interventions (see e.g., Cuijpers et al. 2010; Driessen et al. 2015; Niemeyer et al. 2013). Open science practices, particularly pre‐registrations, registered reports, and open peer reviews, could contribute to making null results more visible. Pre‐registrations can help in finding studies that are not published. Increased transparency in the peer review process, for example, by publishing the reviews themselves, can help to uncover and reduce biases among reviewers and editors (see Salazar Kämpf et al. 2025).

### 4.3 Using Open Science to Move Towards a Positive Error Culture When Dealing With Mistakes

Humans make mistakes. In every profession and at every level of experience. However, in science, mistakes are less welcome than in other areas, as the integrity of the scientific records must be maintained. That means that (at least) everything formally published should be correct. When approaching open science practices, many researchers worry that with increased transparency, colleagues will spot errors or weaknesses in their work, which may result in blame, sanctions, or reduced career prospects (Ehlers and Lonsdorf 2022). In the worst case, this may result in researchers refraining from using open science practices or acknowledging and correcting errors.

We would like to address these worries on three levels. First, by recognizing that humans always make mistakes, we can infer that it is likely there will also be mistakes in our published scientific records. This is all the more true given that analytic procedures grow increasingly complex. A contemporary study in clinical psychology may include elaborate diagnostic procedures, data acquisition using a sophisticated scanning sequence on the fMRI scanner, complex processing pipelines for the resulting data, and a final analysis with cutting‐edge machine learning techniques. Such an analysis pipeline requires immense levels of expertise and includes a multitude of difficult analytical decisions, which may result in more or less severe errors. Adopting open science practices at least allows us to catch these errors and potentially correct them. Thus, if we care about the accuracy of the scientific record, we need to increase our focus on open science practices.

Second, there is the question of the consequences of errors. For the publications concerned, this seems clear in theory: a correction or retraction must be issued, depending on how extensive the errors are and whether or not the core statements of a publication can still be maintained after correction. In practice, however, this seems less clear. On the one hand, there are repeated reports that journals or editors do not react or react only very slowly to notices of errors in publications (Friedman et al. 2020). On the other hand, the supposed consensus also seems less clear among scientists themselves than one might think. Eklund et al. (2016), for example, found a bug in the 3dClustSim analysis software for neuroimaging data that could erroneously lead to significant results. To our knowledge, however, no corrections of affected papers have yet appeared. Additionally, even if a paper is retracted, the original paper, including its erroneous results or material, may still be used as a basis for replications or extensions. Open science platforms offer the opportunity to use the most up‐to‐date (and, if necessary, corrected) versions of material or data. They combine pre‐registrations, material, and data from a project, make them available to the public, and can be continuously updated by the authors (see Salazar Kämpf et al. 2025). In the best case, spotting more errors due to the increased use of open science techniques will lead to a more stringent handling and elimination of such errors.

Among the possible consequences, however, are also concerns many scientists have about blame, sanctions, or reduced career prospects (Abele‐Brehm et al. 2019). Presumably, part of the concern about negative consequences stems from the fact that in the current scientific culture, errors appear to be a rare and exceptional phenomenon. As already mentioned, we are convinced that this is not the case. We are positive that as transparency increases through the increased use of open science techniques, discovering and dealing with errors will become more normalized and lose some of its taboo. This, of course, should not lead to diminished diligence; however, as presumably, concern about being seen as a “sloppy researcher” is another source of worry for many colleagues.

Following this line of thought, the increased use of open science techniques also offers the opportunity to discuss what minimum standards and procedures for error minimization are required and how they can be followed. It is generally assumed that scientists do their best to avoid errors, but what does that entail? Appropriate suggestions or best practice guidelines are only available for some analyses or research fields, and even when they are available, it is often unclear how widely they are disseminated and used. The increased use of open science practices also provides an incentive to increasingly develop minimum standards for minimizing errors and implementing them. An example of this can be open scripts, as they facilitate the reproducibility and replicability of results (Stodden et al. 2018) and speed up similar analyses. Additionally, particularly complex analyses often include steps not fully detailed in the methods section and thus only become clear through the code (for more details, see Salazar Kämpf et al. 2025).

In conclusion, mistakes happen, and this is normal, but mistakes need to be dealt with. This includes identifying and correcting past mistakes and minimizing future mistakes. Particularly when thinking about the development and implementation of new treatment approaches, mistakes may yield catastrophic consequences. If mistakes cannot be dealt with openly and transparently, they are more likely to be brushed under the carpet even when they are spotted, perpetuating potentially unfavorable or even dangerous treatments. Open science practices may normalize dealing with mistakes, are central to their identification, and may provide further incentives for correction and minimization.

### 4.4 Embrace a Multitude of Perspectives to Drive Science Forward…

The strength of science lies in its capacity to integrate the perspectives of all researchers based on the quality of their arguments rather than their social status (Merton 1979). In line with this popular notion, empirical data show that research conducted within diverse teams often has superior results (Campbell et al. 2013), even though working in diverse groups is more challenging (Phillips et al. 2013). In contrast, excluding members of specific groups leads to a loss of expertise, restricting research to the experiences of a homogenous group of scientists. Therefore, from the beginning, one central aspect of the open science idea has been to enable all researchers to access and work with data regardless of, for example, their status, origin, location, and previous achievements.

Clinical psychology, like many other scientific fields, grapples with the persistent issue of underrepresentation, particularly concerning women (Holman et al. 2018), individuals from ethnic minorities (Stewart et al. 2017), and non‐Western academics (Ruiz et al. 2025). In a discipline that seeks to explore the human experience, such limitations are detrimental. Cooperation via open science has the potential to overcome this limitation by way of starting collaborations and promoting scientific exchange, if scientists are open to working in diverse teams. Cooperation through open science can foster inclusivity, harnessing a wealth of perspectives and experiences to enrich the scientific endeavor.

### 4.5 …And the Extent of This Inclusive Attitude Toward Patients as Well

Patient involvement refers to the inclusion of patients—usually referred to as “experts by experience” (Groot et al. 2022)—as participants in the research process, for example, in the selection of research questions, the development of materials, or the interpretation and dissemination of results, rather than passive research subjects (NIHR 2021). The researcher structures this participation and enhances ecological validity and relevance. Since it typically does not imply article writing or statistical analyses, this participation does not necessarily imply co‐authorship. To date, this inclusion has not consistently been considered to be part of open science. However, the involvement of experts by experience should be established as a distinct and relevant aspect of open science, both for research in clinical psychology and the broader context of medical and health services research. This relates to the definition of open science as “transparent and accessible knowledge that is shared and developed through collaborative networks” (Vicente‐Saez and Martinez‐Fuentes 2018, p. 434). Transparency and collaborative work are two focal points of this definition, although they have often been related only to the scientific community. However, we argue that transparency and collaborative working also refer to those affected by our research. Indeed, sources such as the open science definition paper (Vicente‐Saez and Martinez‐Fuentes 2018) and the Open Science Framework's Open Science Concept (Center for Open Science, https://www.cos.io/open-science) already mention citizen science and participatory research as sub‐items of open science. In clinical settings, the involvement of experts with experience should therefore be an equally important aspect of open science in the future, alongside pre‐registration, open data, open materials, and others. Reviews can serve as an introduction and orientation (Arumugam et al. 2023; Brett et al. 2014; Price et al. 2018). Since the implementation of more extensive forms of patient involvement, in particular, is time‐consuming, it should be taken into account in professional recruitment and promotion criteria based on open science practices (Schönbrodt et al. 2022; Gärtner et al. 2022; Fink‐Lamotte 2024). The RESQUE tool, developed by Gärtner et al. (2022), endeavors to quantitatively assess open science engagement at the publication level.

In practice, patient involvement in clinical psychology can take several concrete forms. For example, qualitative exploratory interviews may inform the development of research questions before hypotheses are formalized. Patient advisory boards can contribute to the design of intervention studies, including the selection of outcome measures and the consideration of feasibility. In psychotherapy process research, patients may participate in co‐interpretation workshops to reflect on preliminary findings and ensure that interpretations adequately capture lived experience. In routine clinical settings, feedback from systematic outcome monitoring can inform both clinical care and the refinement of research priorities. Such practices illustrate how participatory approaches can complement methodological rigor rather than replace it.

## 5 Conclusions

### 5.1 Rethinking Science Policy

Open science practices will not be established in clinical psychology overnight, nor will they be established by forcing them on researchers. It is essential to address the concerns of researchers adequately. It is necessary to not just call for open science but also to fund related infrastructures within and across departments in universities and research facilities. Nevertheless, it is also worth examining the current system to see whether it is receptive to open science practices or whether it favors *not* pursuing open science practices. Policy changes could be implemented to support the use of open science practices. For example, the funds provided by public donors for open‐access publications are only a fraction of what an open‐access publication costs today. To create incentives for open science practices, it would be desirable if funding for open‐access publications was significantly increased, thus, allowing prominent publications to be published as open‐access publications. A further incentive would be that the additional effort required for open science practices (e.g., pre‐registration, data processing, and provision) could be supported by additional personnel expenses dedicated specifically to implementing open science. But it is also worth considering an incentive system in clinical psychology, even without funding sources. One possibility is to consider open science practices in doctoral projects. Doctoral regulations in clinical psychology often stipulate that the first author must have two publications, and a third publication must be in preparation to be allowed to submit a cumulative dissertation. Here, alternatively, a publication as the first author, another submitted study as the first author, and pre‐registration, processing, and publication of the data would also be recognized as a third achievement for the cumulative dissertation. Another incentive could be that open science activities are given more significant consideration in appointment procedures for professorships. This is especially pertinent in the field of clinical psychology, where appointments necessitate both clinical expertise and scholarly accomplishments, demanding a judicious allocation of resources. Preliminary results show that the quality of open science activities among early‐career researchers in clinical psychology, as measured by RESQUE, is heterogeneous.

Finally, we would also like to focus more on participatory research. Open science practices, in particular, offer an opportunity to involve patients more in the studies for which they provide their data. For example, a joint decision with patients and researchers could be made regarding the purposes for which the data should be freely available. However, open science practices can also promote interaction between people with lived experiences, practitioners, and researchers. This means that patients and practitioners can contribute to research projects and support researchers in thinking about important aspects of training and care.

In summary, we would emphasize that the scientific system in clinical psychology can be made more receptive to open science practices and that it is desirable if incentives for open science practices are created without, at the same time, creating disadvantages for researchers who decide against using open science practices for their projects.

## Ethics Statement

The authors have nothing to report.

## Conflicts of Interest

The authors declare no conflicts of interest.

## Data Availability

Data sharing not applicable to this article as no datasets were generated or analyzed during the current study.

## References

[jclp70162-bib-0001] Abele‐Brehm, A. E. , M. Gollwitzer , U. Steinberg , and F. D. Schönbrodt . 2019. “Attitudes Toward Open Science and Public Data Sharing.” Social Psychology 50, no. 4: 252–260.

[jclp70162-bib-0083] Acord, S. K. , and D. Harley . 2013. “Credit, Time, and Personality: The Human Challenges to Sharing Scholarly Work Using Web 2.0.” New Media & Society 15, no. 3: 379–397. 10.1177/1461444812465140.

[jclp70162-bib-0003] Alós‐Ferrer, C. , and E. Yechiam . 2020. “At the Eve of the 40th Anniversary of the Journal of Economic Psychology: Standards, Practices, and Challenges.” Journal of Economic Psychology 80: 102309. 10.1016/j.joep.2020.102309.

[jclp70162-bib-0004] Arumugam, A. , L. R. Phillips , A. Moore , et al. 2023. “Patient and Public Involvement in Research: A Review of Practical Resources for Young Investigators.” BMC Rheumatology 7: 2. 10.1186/s41927-023-00327-w. 36895053 PMC9996937

[jclp70162-bib-0005] Bakker, M. , A. van Dijk , and J. M. Wicherts . 2012. “The Rules of the Game Called Psychological Science.” Perspectives on Psychological Science 7, no. 6: 543–554. 10.1177/1745691612459060. 26168111

[jclp70162-bib-0006] Brett, J. , S. Staniszewska , C. Mockford , et al. 2014. “Mapping the Impact of Patient and Public Involvement on Health and Social Care Research: A Systematic Review.” Health Expectations 17: 637–650. 10.1111/j.1369-7625.2012.00795.x. 22809132 PMC5060910

[jclp70162-bib-0007] Brown, S. D. , D. Furrow , D. F. Hill , J. C. Gable , L. P. Porter , and W. J. Jacobs . 2014. “A Duty to Describe: Better the Devil You Know Than the Devil You Don't.” Perspectives on Psychological Science 9, no. 6: 626–640. 10.1177/1745691614551749. 26186113

[jclp70162-bib-0008] Bruchmüller, K. , J. Margraf , A. Suppiger , and S. Schneider . 2011. “Popular or Unpopular? Therapists' Use of Structured Interviews and Their Estimation of Patient Acceptance.” Behavior Therapy 42, no. 4: 634–643. 10.1016/j.beth.2011.02.003. 22035992

[jclp70162-bib-0009] Burghardt, J. , and A. N. Bodansky . 2021. “Why Psychology Needs to Stop Striving for Novelty and How to Move Towards Theory‐Driven Research.” Frontiers in Psychology 12: 609802. 10.3389/fpsyg.2021.609802. 33633639 PMC7902022

[jclp70162-bib-0010] Campbell, L. G. , S. Mehtani , M. E. Dozier , and J. Rinehart . 2013. “Gender‐Heterogeneous Working Groups Produce Higher Quality Science.” PLoS ONE 8, no. 10: e79147. 10.1371/journal.pone.0079147. 24205372 PMC3813606

[jclp70162-bib-0011] Carpenter, T. P. , and K. C. Law . 2021. “Optimizing the Scientific Study of Suicide With Open and Transparent Research Practices.” Suicide and Life‐Threatening Behavior 51, no. 1: 36–46. 10.1111/sltb.12665. 33624871

[jclp70162-bib-0012] Casey . 2021. “Publication Bias: Why Null Results Are Not Necessarily ‘Dull’ Results.” https://www.sydney.edu.au/science/news-and-events/2021/11/22/publication-bias-why-null-results-are-not-necessarily-dull-re.html.

[jclp70162-bib-0014] Chambers, C. D. , and L. Tzavella . 2022. “The Past, Present and Future of Registered Reports.” Nature Human Behaviour 6: 29–42. 10.1038/s41562-021-01193-7. 34782730

[jclp70162-bib-0015] Corman, V. M. , O. Landt , M. Kaiser , et al. 2020. “Detection of 2019 Novel Coronavirus (2019‐nCoV) by Real‐Time RT‐PCR.” Euro Surveillance: Bulletin Europeen Sur Les Maladies Transmissibles = European Communicable Disease Bulletin 25, no. 3: 2000045. 10.2807/1560-7917.ES.2020.25.3.2000045. 31992387 PMC6988269

[jclp70162-bib-0016] Corten, R. 2019. *Social Dilemmas in the Sharing Economy*. 10.31235/osf.io/cq26e.

[jclp70162-bib-0017] Cuijpers, P. 2016. “Are All Psychotherapies Equally Effective in the Treatment of Adult Depression? The Lack of Statistical Power of Comparative Outcome Studies.” Evidence Based Mental Health 19, no. 2: 39–42. 10.1136/eb-2016-102341. 26984413 PMC10699414

[jclp70162-bib-0018] Cuijpers, P. , F. Smit , E. Bohlmeijer , S. D. Hollon , and G. Andersson . 2010. “Efficacy of Cognitive–Behavioural Therapy and Other Psychological Treatments for Adult Depression: Meta‐Analytic Study of Publication Bias.” British Journal of Psychiatry 196, no. 3: 173–178. 10.1192/bjp.bp.109.066001. 20194536

[jclp70162-bib-0019] Curry, S. , S. de Rijcke , A. Hatch , D. Pillay , I. van der Weijden , and J. Wilsdon . 2020. “The Changing Role of Funders in Responsible Research Assessment: Progress, Obstacles and the Way Ahead.” 10.6084/m9.figshare.13227914.v1.

[jclp70162-bib-0020] Cwik, J. C. , and T. Teismann . 2017. “Misclassification of Self‐Directed Violence.” Clinical Psychology & Psychotherapy 24, no. 3: 677–686. 10.1002/cpp.2036. 27481725

[jclp70162-bib-0081] Doroshow, J. H. , and S. Kummar . 2014. “Translational Research in Oncology–10 Years of Progress and Future Prospects.” Nature Reviews. Clinical Oncology 11, no. 11: 649–662. 10.1038/nrclinonc.2014.158. 25286976

[jclp70162-bib-0021] Driessen, E. , S. D. Hollon , C. L. H. Bockting , P. Cuijpers , and E. H. Turner . 2015. “Does Publication Bias Inflate the Apparent Efficacy of Psychological Treatment for Major Depressive Disorder? A Systematic Review and Meta‐Analysis of US National Institutes of Health‐Funded Trials.” PLoS ONE 10, no. 9: e0137864. 10.1371/journal.pone.0137864. 26422604 PMC4589340

[jclp70162-bib-0022] Ehlers, M. R. , and T. B. Lonsdorf . 2022. “Data Sharing in Experimental Fear and Anxiety Research: From Challenges to a Dynamically Growing Database in 10 Simple Steps.” Neuroscience and Biobehavioral Reviews 143: 104958. 10.1016/j.neubiorev.2022.104958. 36372236

[jclp70162-bib-0023] Eklund, A. , T. E. Nichols , and H. Knutsson . 2016. “Cluster Failure: Why fMRI Inferences for Spatial Extent Have Inflated False‐Positive Rates.” Proceedings of the National Academy of Sciences 113, no. 28: 7900–7905. 10.1073/pnas.160241311. PMC4948312 27357684

[jclp70162-bib-0025] Fink‐Lamotte, J. , K. Hilbert , D. Bentz , et al. 2024. “Response to Responsible Research Assessment I and II From the Perspective of the DGPs Working Group on Open Science in Clinical Psychology.” Meta‐Psychology 8. 10.15626/mp.2023.3794.

[jclp70162-bib-0026] Friedman, H. L. , D. A. MacDonald , and J. C. Coyne . 2020. “Working With Psychology Journal Editors to Correct Problems in the Scientific Literature.” Canadian Psychology/Psychologie Canadienne 61, no. 4: 342–348. 10.1037/cap0000248.

[jclp70162-bib-0027] Gärtner, A. , D. Leising , and F. D. Schönbrodt . 2022. “Responsible Research Assessment II: A Specific Proposal for Hiring and Promotion in Psychology.” 10.31234/osf.io/5yexm.

[jclp70162-bib-0028] Gold, E. R. 2021. “The Fall of the Innovation Empire and Its Possible Rise Through Open Science.” Research Policy 50, no. 5: 104226. 10.1016/j.respol.2021.104226. 34083844 PMC8024784

[jclp70162-bib-0029] Groot, B. , A. Haveman , M. Buree , R. Zuijlen , J. Zuijlen , and T. Abma . 2022. “What Patients Prioritize for Research to Improve Their Lives and How Their Priorities Get Dismissed Again.” International Journal of Environmental Research and Public Health 19, no. 4: 1927. 10.3390/ijerph19041927. 35206113 PMC8871903

[jclp70162-bib-0085] Hardy, C. L. , and M. Van Vugt . 2006. “Nice Guys Finish First: The Competitive Altruism Hypothesis.” Personality & Social Psychology Bulletin 32, no. 10: 1402–1413. 10.1177/0146167206291006. 16963610

[jclp70162-bib-0082] Hesse, B. W. 2018. “Can Psychology Walk the Walk of Open Science?” American Psychologist 73, no. 2: 126–137. 10.1037/amp0000197. 29481106

[jclp70162-bib-0031] Holman, L. , D. Stuart‐Fox , and C. E. Hauser . 2018. “The Gender Gap in Science: How Long Until Women Are Equally Represented?” PLoS Biology 16, no. 4: e2004956. 10.1371/journal.pbio.2004956. 29672508 PMC5908072

[jclp70162-bib-0032] Iwachiw, J. S. , A. L. Button , and J. Atlas . 2019. “The Perceived Role of Null Results in School Psychology Research and Publication.” School Psychology International 40, no. 4: 416–430. 10.1177/0143034319851230.

[jclp70162-bib-0033] Kirtley, O. J. , J. J. Janssens , and A. Kaurin . 2022. “Open Science in Suicide Research Is Open for Business.” Crisis 43, no. 5: 355–360. 10.1027/0227-5910/a000859. 35915973 PMC9645435

[jclp70162-bib-0035] Lakens, D. , C. Mesquida , S. Rasti , and M. Ditroilo . 2024. “The Benefits of Preregistration and Registered Reports.” Evidence‐Based Toxicology 2, no. 1: 2376046. 10.1080/2833373X.2024.2376046.

[jclp70162-bib-0036] Langham‐Putrow, A. , C. Bakker , and A. Riegelman . 2021. “Is the Open Access Citation Advantage Real? A Systematic Review of the Citation of Open Access and Subscription‐Based Articles.” PLoS ONE 16, no. 6: e0253129. 10.1371/journal.pone.0253129. 34161369 PMC8221498

[jclp70162-bib-0038] Leichsenring, F. , A. Abbass , M. J. Hilsenroth , et al. 2017. “Biases in Research: Risk Factors for Non‐Replicability in Psychotherapy and Pharmacotherapy Research.” Psychological Medicine 47, no. 6: 1000–1011. 10.1017/S003329171600324X. 27955715

[jclp70162-bib-0039] Lincoln, T. M. , L. Schulze , and B. Renneberg . 2022. “The Role of Emotion Regulation in the Characterization, Development and Treatment of Psychopathology.” Nature Reviews Psychology 1, no. 5: 272–286. 10.1038/s44159-022-00040-4.

[jclp70162-bib-0040] Linek, S. B. , B. Fecher , S. Friesike , and M. Hebing . 2017. “Data Sharing as Social Dilemma: Influence of the Researcher's Personality.” PLoS ONE 12, no. 8: e0183216. 10.1371/journal.pone.0183216. 28817642 PMC5560561

[jclp70162-bib-0041] Margraf, J. , J. C. Cwik , V. Pflug , and S. Schneider . 2017. “Strukturierte Klinische Interviews Zur Erfassung Psychischer Störungen Über Die Lebensspanne: Gütekriterien Und Weiterentwicklungen Der DIPS‐Verfahren.” Zeitschrift für Klinische Psychologie und Psychotherapie 46, no. 3: 176–186. 10.1026/1616-3443/a000430.

[jclp70162-bib-0042] Merton, R. K. 1979. The Sociology of Science: Theoretical and Empirical Investigations, edited by N. W. Storer . Chicago, IL: University of Chicago Press.

[jclp70162-bib-0043] Moffitt, T. E. , A. Caspi , A. Taylor , et al. 2010. “How Common Are Common Mental Disorders? Evidence That Lifetime Prevalence Rates Are Doubled by Prospective *Versus* Retrospective Ascertainment.” Psychological Medicine 40, no. 6: 899–909. 10.1017/S0033291709991036. 19719899 PMC3572710

[jclp70162-bib-0044] Moshontz, H. , L. Campbell , C. R. Ebersole , et al. 2018. “The Psychological Science Accelerator: Advancing Psychology Through a Distributed Collaborative Network.” Advances in Methods and Practices in Psychological Science 1, no. 4: 501–515. 10.1177/2515245918797607. 31886452 PMC6934079

[jclp70162-bib-0045] Murphy, K. R. , and H. Aguinis . 2019. “HARKing: How Badly Can Cherry‐Picking and Question Trolling Produce Bias in Published Results?” Journal of Business and Psychology 34, no. 1: 1–17. 10.1007/s10869-017-9524-7.

[jclp70162-bib-0046] Niemeyer, H. , J. Musch , and R. Pietrowsky . 2013. “Publication Bias in Meta‐Analyses of the Efficacy of Psychotherapeutic Interventions for Depression.” Journal of Consulting and Clinical Psychology 81, no. 1: 58–74. 10.1037/a0031152. 23244368

[jclp70162-bib-0047] Niemeyer, H. , C. Knaevelsrud , R. C. M. van Aert , and T. Ehring . 2023. “Research into Evidence‐Based Psychological Interventions Needs a Stronger Focus on Replicability.” Clinical Psychology in Europe 5, no. 3: e9997. 10.32872/cpe.9997. 38356898 PMC10863633

[jclp70162-bib-0048] NIHR . “Briefing Notes for Researchers, April 2021.” Accessed February 1, 2024. https://www.nihr.ac.uk/documents/briefing-notes-for-researchers-public-involvement-in-nhs-health-and-social-care-research/27371.

[jclp70162-bib-0049] Nuijten, M. B. , C. H. J. Hartgerink , M. A. L. M. van Assen , S. Epskamp , and J. M. Wicherts . 2016. “The Prevalence of Statistical Reporting Errors in Psychology (1985–2013).” Behavior Research Methods 48, no. 4: 1205–1226. 10.3758/s13428-015-0664-2. 26497820 PMC5101263

[jclp70162-bib-0051] Petersen, I. T. , K. S. Apfelbaum , and B. McMurray . 2024. “Adapting Open Science and Pre‐Registration to Longitudinal Research.” Infant and Child Development 33: e2315. 10.1002/icd.2315. 38425545 PMC10904029

[jclp70162-bib-0052] Phillips, K. W. , M. Duguid , M. Thomas‐Hunt , and J. Uparna . 2013. *Diversity as Knowledge Exchange: The Roles of Information Processing, Expertise, and Status (Q. M. Roberson, Hrsg.)*. Oxford University Press. https://www.oxfordhandbooks.com/view/10.1093/oxfordhb/9780199736355.001.0001/oxfordhb-9780199736355-e-9.

[jclp70162-bib-0053] Price, A. , L. Albarqouni , J. Kirkpatrick , et al. 2018. “Patient and Public Involvement in the Design of Clinical Trials: An Overview of Systematic Reviews.” Journal of Evaluation in Clinical Practice 24, no. 1: 240–253. 29076631 10.1111/jep.12805

[jclp70162-bib-0054] Reardon, K. W. , A. J. Smack , K. Herzhoff , and J. L. Tackett . 2019. “An N‐Pact Factor for Clinical Psychological Research.” Journal of Abnormal Psychology 128, no. 6: 493–499. 10.1037/abn0000435. 31368728

[jclp70162-bib-0086] Richter, G. , N. Trigui , A. Caliebe , and M. Krawczak . 2024. “Attitude Towards Consent‐Free Research Use of Personal Medical Data in the General German Population.” Heliyon 10, no. 6. 10.1016/j.heliyon.2024.e27933. PMC10951576 38509969

[jclp70162-bib-0055] Rockhold, F. , C. Bromley , E. K. Wagner , and M. Buyse . 2019. “Open Science: The Open Clinical Trials Data Journey.” Clinical Trials 16, no. 5: 539–546. 10.1177/1740774519865512. 31347390

[jclp70162-bib-0056] Rose, S. , J. Bisson , and S. Wessely . 2003. “A Systematic Review of Single‐Session Psychological Interventions (‘Debriefing’) Following Trauma.” Psychotherapy and Psychosomatics 72, no. 4: 176–184. 10.1159/000070781. 12792122

[jclp70162-bib-0088] Ruiz, N. , M. Gallagher , and R. Najjuma . 2025. “Postdigital Science and Education and The Majority World.” Postdigital Science and Education. 10.1007/s42438-025-00545-0.

[jclp70162-bib-0057] Salazar Kämpf, M. , M. Riehle , J. C. Ehrenthal , et al. 2025. “How to Practice Open Science: A Cookbook for Clinical Psychology Research.” 10.31234/osf.io/ztvjm_v1.

[jclp70162-bib-0059] Schönbrodt, F. D. , A. Gärtner , M. Frank , et al. 2022. “Responsible Research Assessment I: Implementing DORA for Hiring and Promotion in Psychology.” 10.31234/osf.io/rgh5b.

[jclp70162-bib-0061] Shrout, P. E. , and J. L. Rodgers . 2018. “Psychology, Science, and Knowledge Construction: Broadening Perspectives From the Replication Crisis.” Annual Review of Psychology 69, no. 1: 487–510. 10.1146/annurev-psych-122216-011845. 29300688

[jclp70162-bib-0062] Simmons, J. P. , L. D. Nelson , and U. Simonsohn . 2011. “False‐Positive Psychology: Undisclosed Flexibility in Data Collection and Analysis Allows Presenting Anything as Significant.” Psychological Science 22, no. 11: 1359–1366. 10.1177/0956797611417632. 22006061

[jclp70162-bib-0063] Stodden, V. , J. Seiler , and Z. Ma . 2018. “An Empirical Analysis of Journal Policy Effectiveness for Computational Reproducibility.” Proceedings of the National Academy of Sciences 115, no. 11: 2584–2589. 10.1073/pnas.1708290115. PMC5856507 29531050

[jclp70162-bib-0087] Stewart, C. E. , S. Y. Lee , A. Hogstrom , and M. Williams . 2017. “Diversify and Conquer: A Call to Promote Minority Representation in Clinical Psychology.” Behavior Therapist 40, no. 3: 74–79.

[jclp70162-bib-0064] Tackett, J. L. , S. O. Lilienfeld , C. J. Patrick , et al. 2017. “It's Time to Broaden the Replicability Conversation: Thoughts for and From Clinical Psychological Science.” Perspectives on Psychological Science 12, no. 5: 742–756. 10.1177/1745691617690042. 28972844

[jclp70162-bib-0065] Tackett, J. L. , C. M. Brandes , and K. W. Reardon . 2019. “Leveraging the Open Science Framework in Clinical Psychological Assessment Research.” Psychological Assessment 31, no. 12: 1386–1394. 10.1037/pas0000583. 30869959

[jclp70162-bib-0066] Tackett, J. L. , C. M. Brandes , K. M. King , and K. E. Markon . 2019. “Psychology's Replication Crisis and Clinical Psychological Science.” Annual Review of Clinical Psychology 15, no. 1: 579–604. 10.1146/annurev-clinpsy-050718-095710. 30673512

[jclp70162-bib-0084] Tenopir, C. , S. Allard , K. Douglass , et al. 2011. “Data Sharing by Scientists: Practices and Perceptions.” PLOS ONE 6, no. 6: e21101. 10.1371/journal.pone.0021101. 21738610 PMC3126798

[jclp70162-bib-0067] Trespidi, C. , C. Barbui , and A. Cipriani . 2011. “Why It Is Important to Include Unpublished Data in Systematic Reviews.” Epidemiology and Psychiatric Sciences 20, no. 2: 133–135. 10.1017/S2045796011000217. 21714359

[jclp70162-bib-0068] Trespidi, C. , C. Barbui , and A. Cipriani . 2011. “Why It Is Important to Include Unpublished Data in Systematic Reviews.” Epidemiology and Psychiatric Sciences 20, no. 2: 133–135. 10.1017/s2045796011000217. 21714359

[jclp70162-bib-0069] United Nations Educational, Scientific and Cultural Organization . 2021. “UNESCO Recommendation on Open Science (SC‐PCB‐SPP/2021/OS/UROS.” pp. 1–34. https://unesdoc.unesco.org/ark:/48223/pf0000379949.locale=en.

[jclp70162-bib-0070] Vicente‐Saez, R. , and C. Martinez‐Fuentes . 2018. “Open Science Now: A Systematic Literature Review for an Integrated Definition.” Journal of Business Research 88: 428–436. 10.1016/j.jbusres.2017.12.043.

[jclp70162-bib-0071] Wagenmakers, E. J. , and G. Dutilh . 2016. “Seven Selfish Reasons for Preregistration.” APS Observer 29: 13–14.

[jclp70162-bib-0072] Walsh, C. G. , W. Xia , M. Li , J. C. Denny , P. A. Harris , and B. A. Malin . 2018. “Enabling Open‐Science Initiatives in Clinical Psychology and Psychiatry Without Sacrificing Patients' Privacy: Current Practices and Future Challenges.” Advances in Methods and Practices in Psychological Science 1, no. 1: 104–114. 10.1177/2515245917749652.

[jclp70162-bib-0073] Whittington, C. J. , T. Kendall , P. Fonagy , D. Cottrell , A. Cotgrove , and E. Boddington . 2004. “Selective Serotonin Reuptake Inhibitors in Childhood Depression: Systematic Review of Published Versus Unpublished Data.” The Lancet 363, no. 9418: 1341–1345. 10.1016/S0140-6736(04)16043-1. 15110490

[jclp70162-bib-0074] Wolkenstein, L. , K. Bruchmüller , P. Schmid , and T. D. Meyer . 2011. “Misdiagnosing Bipolar Disorder ‐ Do Clinicians Show Heuristic Biases?” Journal of Affective Disorders 130, no. 3: 405–412. 10.1016/j.jad.2010.10.036. 21093059

[jclp70162-bib-0075] Youyou, W. , Y. Yang , and B. Uzzi . 2023. “A Discipline‐Wide Investigation of the Replicability of Psychology Papers over the Past Two Decades.” Proceedings of the National Academy of Sciences 120, no. 6: e2208863120. 10.1073/pnas.2208863120. PMC9963456 36716367

